# Non-Erythropoietic EPO (EPO-R76E) Protects RPE Cells from Ferroptosis by Modulating the Labile Iron Pool and NRF2-GPX4 Axis †

**DOI:** 10.3390/antiox15050647

**Published:** 2026-05-20

**Authors:** Sundaramoorthy Gopi, George T. Prodanoff, Christopher L. Passaglia, Mark S. Kindy, Vijaykumar Sutariya, Ganesh V. Halade, Alfred S. Lewin, Manas R. Biswal

**Affiliations:** 1 Department of Pharmaceutical Sciences, Taneja College of Pharmacy, University of South Florida, Tampa, FL 33612, USA; gopis@usf.edu (S.G.); gprodanoff@usf.edu (G.T.P.); kindym@usf.edu (M.S.K.); vsutariy@usf.edu (V.S.); 2 Department of Medical Engineering and College of Engineering and Morsani College of Medicine, University of South Florida, Tampa, FL 33612, USA; passaglia@usf.edu; 3 Research Service, James Haley VA Medical Center, Tampa, FL 33612, USA; 4 Heart Institute, Division of Cardiovascular Sciences, Department of Internal Medicine, Morsani College of Medicine, University of South Florida, Tampa, FL 33612, USA; ghalade@usf.edu; 5 Hypertension and Kidney Research Center, University of South Florida, Tampa, FL 33612, USA; 6 Department of Molecular Genetics & Microbiology, University of Florida, Gainesville, FL 32608, USA; lewin@ufl.edu; 7 Department of Ophthalmology, University of Florida, Gainesville, FL 32608, USA; 8 Department of Ophthalmology, Morsani College of Medicine, University of South Florida, Tampa, FL 33612, USA; 9 Department of Internal Medicine, Morsani College of Medicine, University of South Florida, Tampa, FL 33612, USA

**Keywords:** age-related macular degeneration (AMD), retinal pigment epithelium (RPE), gene therapy, ferroptosis, lipid peroxidation, iron homeostasis, ferric ammonium citrate (FAC), labile iron pool, neuroprotection, NRF2 signaling, GPX4, p62/SQSTM1, LC3B, oxidative stress, modified variant of erythropoietin, Epo-R76E, antioxidants, autophagy

## Abstract

Retinal pigment epithelium (RPE) degeneration remains a formidable challenge in dry age-related macular degeneration (AMD) research, primarily due to the toxic interplay between iron overload and ferroptosis. We investigated whether EPO-R76E, a non-erythropoietic modified variant of erythropoietin, could effectively interrupt this destructive cycle. Using ARPE-19 cells challenged with ferric ammonium citrate (FAC) to model iron-induced toxicity, we show that EPO-R76E confers protection against ferroptosis. Our results demonstrate that this variant significantly reduces the intracellular labile iron pool, directly quenching the lipid peroxidation that drives ferroptotic cell death. This resilience is fueled by a robust upregulation of Glutathione Peroxidase 4 (GPX4) and the broad transcriptional activation of the NRF2 (Nuclear factor erythroid 2-related factor 2) NRF2 antioxidant axis. Furthermore, we found that EPO-R76E enhances autophagic flux, ensuring that cells maintain essential proteostasis and “housekeeping” functions even under metabolic crisis. By integrating iron sequestration with reinforced antioxidant signaling and cellular clearing mechanisms, EPO-R76E stands out as a potent candidate for preserving RPE health. These findings uncover a novel molecular framework for protecting the retina against iron-mediated injury, positioning EPO-R76E as a versatile and targeted gene-based therapeutic for addressing the fundamental causes of retinal degeneration.

## 1. Introduction

Age-related macular degeneration (AMD) is a progressive neurodegenerative disease and remains the leading cause of irreversible vision loss in individuals over 50 years of age in developed countries [1]. This multifactorial disorder primarily affects the macula, the central region of the retina responsible for high-resolution vision, resulting in significant visual impairment and a profound reduction in quality of life [2]. The pathogenesis of AMD is notoriously complex, involving a delicate interplay of genetic predisposition, environmental influences, and age-related metabolic shifts. At the core of this degeneration lies the dysfunction of the retinal pigment epithelium (RPE), a specialized monolayer of cells essential for photoreceptor survival [3]. Structural alterations, including the accumulation of lipofuscin, the formation of sub-RPE deposits known as drusen, and progressive thickening of Bruch’s membrane, collectively contribute to a toxic microenvironment that precipitates RPE death and subsequent vision loss [4]. Beyond traditional models of apoptosis, recent evidence has identified ferroptosis, a regulated, iron-dependent form of cell death, as a primary executioner of the RPE in AMD [5]. Ferroptosis is driven by the catastrophic accumulation of lipid hydroperoxides resulting from the failure of glutathione-dependent antioxidant defenses, most notably the inactivation of Glutathione Peroxidase 4 (GPX4) [6]. The RPE is uniquely susceptible to this pathway due to its high oxygen consumption, dense mitochondrial population, and elevated concentration of polyunsaturated fatty acids (PUFAs) in its membranes [7]. Lipidomic studies have revealed that oxidized phosphatidylethanolamines and specific phospholipids serve as the primary substrates for iron-induced lipid peroxidation in RPE cells. The resulting accumulation of toxic byproducts, such as 4-hydroxynonenal (4-HNE) and malondialdehyde (MDA), exacerbates mitochondrial dysfunction and disrupts the homeostatic relationship between the RPE and the neural retina [8].

Recent mechanistic insights have further highlighted the role of ACSL4 (Acyl-CoA Synthetase Long-Chain Family Member 4) and 15-lipoxygenase in amplifying ferroptotic lipid peroxidation, distinguishing this process from other regulated cell death pathways in AMD models [9]. Advanced imaging of patient-derived RPE cells has revealed that ferroptotic cells exhibit distinct mitochondrial shrinkage and increased membrane density prior to the onset of terminal cell death [10]. Furthermore, evidence from *in vivo* AMD models demonstrates that enhancing GPX4 activity, either genetically or pharmacologically, can effectively mitigate lipid peroxidation and protect the retinal architecture [11]. Conversely, the loss of ferroptosis suppressor protein 1 (FSP1) or the depletion of glutathione renders the RPE significantly more vulnerable to iron-induced lethality [12]. Given the central role of iron overload and disrupted lipid metabolism in the dry form of AMD, targeting the ferroptotic cascade has emerged as a promising therapeutic strategy to preserve RPE integrity and prevent the progression of geographic atrophy [13].

In the search for pleiotropic cytoprotective agents, Erythropoietin (EPO) has garnered significant attention for its potent neuroprotective, antioxidant, and anti-inflammatory properties in ocular diseases [14]. Classically recognized for its role in erythropoiesis, EPO has been shown to preserve photoreceptors against light-induced damage and support retinal ganglion cell survival in preclinical models of glaucoma and retinitis pigmentosa [15]. These protective effects are mediated through the scavenging of reactive oxygen species (ROS), the induction of endogenous antioxidant defenses, and the activation of pro-survival signaling pathways such as the PI3K/Akt axis [16]. Furthermore, the ability of EPO to cross the blood-retinal barrier and exert localized effects makes it a compelling candidate for retinal therapy [17].

However, the clinical translation of native EPO is severely limited by its hematopoietic side effects, including increased erythrocyte production and thromboembolic risk, particularly for chronic treatments required in retinal degenerations [18]. To overcome these limitations, non-erythropoietic modified variants of EPO such as EPO-R76E have been developed. EPO-R76E features a strategic arginine-to-glutamic acid substitution that eliminates its affinity for the erythropoietic homodimeric receptor (EPOR) while retaining its ability to bind the tissue-protective heteroreceptor complex (EPOR/CD131), also known as the Innate Repair Receptor [19]. This variant has demonstrated a superior safety profile compared to wild-type EPO while providing equivalent or enhanced cytoprotection in sensitive tissues. Our research group has established the robust efficacy of this modified variant across multiple models of RPE stress. We recently demonstrated that EPO-R76E gene therapy effectively protects ARPE-19 cells from acute oxidative damage induced by the toxin Paraquat [20]. Moreover, we validated the translational potential of this variant in an *in vivo* paradigm of chronic mitochondrial stress, where EPO-R76E significantly delayed retinal degeneration in mice with an RPE-specific superoxide dismutase 2 (Sod2) deletion [21]. These studies highlight the ability of EPO-R76E to activate the NRF2/ Heme oxygenase-1 (HO-1) pathway and reduce lipid peroxidation in the face of severe redox imbalance [22]. Despite these advancements, the specific role of EPO-R76E in modulating the biochemical hallmarks of ferroptosis, namely the labile iron pool (LIP) and autophagic quality control, remains to be fully elucidated.

The rationale for the current study is based on the critical need to identify multi-targeted therapies that can intercept the intersecting pathways of iron overload, lipid peroxidation, and autophagic failure in the aging RPE. While native EPO is a potent survival signal, its potential to stimulate subretinal neovascularization via Vascular endothelial growth factor (VEGF) independent pathways is a significant clinical deterrent in AMD patients [23]. By utilizing the non-erythropoietic EPO-R76E variant, we aim to provide a safer and more targeted therapeutic approach. ARPE-19 cells, a widely accepted in vitro model for human RPE, provides a rigorous platform for investigating the protective effects of EPO-R76E against ferroptosis induced by FAC [24].

This study is designed to explore the molecular mechanisms by which EPO-R76E preserves RPE cells when challenged with iron-induced oxidative stress. Specifically, we investigate whether stable expression of EPO-R76E can attenuate LIP, bolster the NRF2-mediated antioxidant axis, and restore autophagic flux as a secondary defense mechanism. By understanding these multifaceted pathways, we hope to uncover new therapeutic targets and establish EPO-R76E as a promising candidate for gene therapy aimed at mitigating vision loss associated with age-related macular degeneration and other iron-associated retinal dystrophies.

## 2. Materials and Methods

### 2.1. Cell Culture and Generation of Stable EPO-R76E-Expressing Lines

The ARPE-19 human retinal pigment epithelium (RPE) cell line was utilized as an in vitro model due to its capacity to exhibit key physiological characteristics of native RPE, making it a standard platform for investigating AMD-related pathologies [25]. Cells were maintained in DMEM/F12 medium supplemented with 10% fetal bovine serum (FBS) and 1% penicillin-streptomycin at 37 °C in a 5% CO_2_ environment. ARPE-19 cells were used between passages 3 to 11, and all experiments were conducted within this defined passage window to minimize phenotypic variability and ensure reproducibility. To evaluate the cytoprotective effects of the non-erythropoietic variant, we utilized stable ARPE-19 lines expressing EPO-R76E (St-EPO), previously generated and validated by our group [20]. The bicistronic lentiviral vector was designed under the control of an elongation factor 1-alpha promoter (EF1) promoter, containing the human EPO-R76E coding sequence followed by a self-cleaving T2A peptide and a Puromycin resistance gene (pac). This architecture utilizes ribosomal skipping to ensure the stoichiometric co-expression of the therapeutic cargo and the selection marker from a single transcript [26]. Transduced cells were selected using Puromycin (2 µg/mL) for 7 days to establish a homogenous stable population. Successful integration and sustained expression were re-validated for the current study via Western blot for the ~26 kDa EPO-R76E protein, with α-tubulin (~51 kDa) serving as the internal loading control. Additionally, transcriptional upregulation was confirmed via Reverse Transcription–quantitative Polymerase Chain Reaction (RT-qPCR) to ensure the stability of the transgene over multiple passages.

### 2.2. Induction of Ferroptosis via Iron Overload

To simulate the iron-rich microenvironment of the aging retina [27], ferroptosis was induced using FAC. FAC is a documented iron donor that expands the intracellular labile iron pool, thereby catalyzing lipid peroxidation and membrane rupture. Cells were seeded at a density of 1 × 10^5^ cells/well in 6-well plates. After 24 h of attachment, cells were transitioned to serum-free medium and treated with FAC (500–2000 µM) for 24 h. FAC was used at a concentration selected based on prior studies in ARPE-19 cells demonstrating reliable induction of iron-associated oxidative stress while preserving sufficient cell viability for downstream analyses. Serum-free conditions were maintained during treatment to prevent the unintended chelation of FAC by serum proteins, ensuring a reproducible induction of iron-dependent stress.

### 2.3. Cell Viability and Flow Cytometry

Metabolic activity was quantified using the WST-1 assay (Roche Diagnostics, Indianapolis, IN, USA). Cells were seeded in 96-well plates (1 × 10^4^ cells/well) and treated with FAC (0–500 µM). Following 24 h of exposure, WST-1 reagent was added for 3 h, and absorbance was measured at 450 nm. To specifically assess terminal cell death and membrane compromise, flow cytometry was performed using propidium iodide (PI) exclusion. Cells were detached, resuspended in binding buffer, and incubated with 5 µL PI (Bio-Rad, Hercules, CA, USA) for 20 min. A total of 20,000 events per sample were analyzed using a BD FACSCanto system to determine the percentage of dead cells in parental vs. St-EPO populations.

### 2.4. Assessment of Lipid Peroxidation and ROS Accumulation

Lipid peroxidation, a definitive hallmark of ferroptosis, was visualized using the Click-iT™ Lipid Peroxidation Imaging Kit (Thermo Fisher, Waltham, MA, USA). Cells were incubated with 50 µM linoleamide alkyne (LAA) for 30 min at 37 °C to allow integration into cellular membranes. Following FAC challenge, oxidized lipids were fluorescently labeled via a copper-catalyzed “click” reaction. Total intracellular reactive oxygen species (ROS) were measured using DCFDA (2′,7′-dichlorofluorescin diacetate). Cells were loaded with 10 µM DCFDA for 30 min in the dark, and fluorescence intensity was captured using a Keyence BZX 800 microscope, Keyence Corporation, Osaka, Japan. All images were quantified using ImageJ software (https://imagej.net/ij/, accessed on 14 April 2026), analyzing at least five random fields across three independent biological replicates.

### 2.5. Intracellular Ferrous Ion (Fe^2+^) Quantification

Dynamic changes in the LIP were quantified using the Fe^2+^-specific fluorescent probe Ferro Orange (Cell Signaling Technology, Danvers, MA, USA). Cells were seeded in 12-well plates at a density of 5 × 10^4^ cells per well and treated with 500 µM FAC. Following treatment, cells were washed with PBS and incubated with 1 µM Ferro-Orange in serum-free medium for 30 min at 37 °C. Fluorescence images (Ex/Em: 543/580 nm) were acquired immediately using a Keyence BZX-800 fluorescence microscope to minimize photobleaching. Quantitative analysis of intracellular Fe^2+^ levels was performed by measuring mean fluorescence intensity using ImageJ software.

### 2.6. Protein Expression Analysis (Western Blotting)

Total protein was extracted using RIPA buffer supplemented with protease inhibitors. Protein concentrations were normalized using the Pierce™ 660 nm assay. Equal amounts of protein (20 µg) were resolved on NuPAGE™ 4–12% Bis-Tris gels and transferred to PVDF or nitrocellulose membranes. Membranes were blocked with Intercept^®^ (PBS) buffer and incubated overnight at 4 °C with primary antibodies against Ferritin (1:5000; MA5-32244), Sequestosome-1 (SQSTM1/p62) (1:10,000; ab109012), GPX4 (1:1000; 67763-1-Ig), Microtubule-associated protein 1 light chain 3B (LC3B) (Ratio, source, Cat no) and β-Actin (1:2000; SC-47778). Signals were detected using IRDye^®^ secondary antibodies and imaged on the iBright Imaging System, Waltham, MA, USA. Densitometric analysis was performed using ImageJ software, and protein expression levels were normalized to β-actin.

### 2.7. Quantitative Real-Time PCR (RT-qPCR)

Total RNA was isolated using the RNeasy Mini Kit (Qiagen, Hilden, North Rhine-Westphalia, Germany) and reverse-transcribed into cDNA (iScript™, Bio-Rad). Quantitative PCR was conducted using SYBR Green Master Mix and gene-specific primers (Table 1) for antioxidant and autophagic targets, including NRF2 (Gene ID: 4780; RefSeq: NM_006164), Catalase (CAT) (Gene ID: 847; RefSeq: NM_001752), NAD(P)H quinone dehydrogenase 1 (NQO1) (Gene ID: 1728; RefSeq: NM_000903), Heme oxygenase-1 (*HO*-1) (Gene ID: 3162; RefSeq: NM_002133), Glutathione S-Transferase Mu 1 (GSTM1) (Gene ID: 2944; RefSeq: NM_000561), and SQSTM1 (Gene ID: 8878; RefSeq: NM_003900). We employed 2^ΔΔCt^ method to determine relative fold changes, with β-actin (Gene ID: 60; RefSeq: NM_001101) serving as the internal reference gene. Primer sequences were verified for gene specificity and optimal amplification efficiency.

All in vitro experiments were performed using at least three independent biological replicates (*n* = 3), unless otherwise stated. Each biological replicate included 2–3 technical replicates per condition.

### 2.8. Statistical Analysis

Statistical analyses were performed using GraphPad Prism 5.0. Differences between experimental groups were analyzed using two-tailed Student’s *t*-tests or One-way ANOVA with Tukey’s post hoc test was used for multiple comparisons, as specified in the figure legends. Data are expressed as mean ± standard error of the mean (SEM). A *p*-value of <0.05 was considered statistically significant (* *p* < 0.05, ** *p* < 0.01, *** *p* < 0.001, **** *p* < 0.0001).

## 3. Results

### 3.1. EPO-R76E Enhances RPE Resilience to Iron-Induced Cytotoxicity

To investigate the therapeutic potential of the non-erythropoietic variant EPO-R76E, we utilized a stable ARPE-19 cell line (St-EPO) previously validated in our investigation of paraquat-induced oxidative stress [20]. The bicistronic expression construct, featuring an EF1 promoter driving EPO-R76E and a Puromycin resistance gene separated by a self-cleaving T2A peptide (Supplementary Figure S1A), was re-validated to ensure sustained transgene expression. Immunoblotting confirmed the production of the ~26 kDa EPO-R76E protein, with α-tubulin (~51 kDa) serving as the loading control (Supplementary Figure S1B,C). Significant transcriptional upregulation of EPO in the St-EPO lines was further confirmed via RT-qPCR compared to parental controls (Supplementary Figure S1D). ARPE-19 cells were selected as a validated in vitro model for studying RPE oxidative stress and ferroptosis pathways relevant to AMD [28,29]. Following this verification of stable expression, we challenged the cells with FAC to simulate the chronic iron overload characteristic of the aging subretinal space.

**Figure 1:**
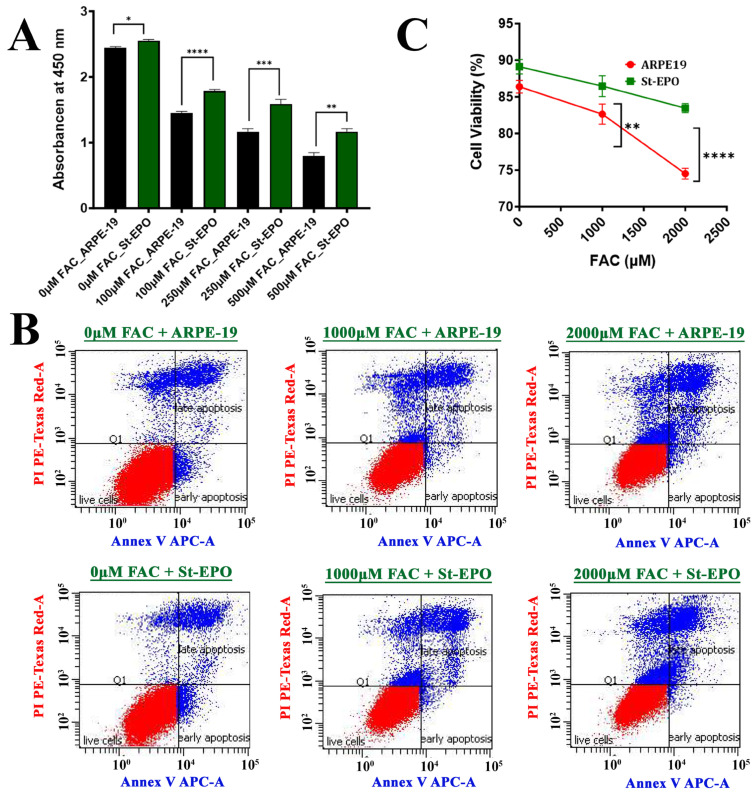
EPO-R76E confers cytoprotection against FAC-induced ferroptotic cell death in ARPE-19 cells. (**A**) Assessment of cell viability via WST-1 assay. Parental ARPE-19 and EPO-R76E-stably expressing (St-EPO) cells were treated with increasing concentrations of FAC (0, 100, 250, and 500 µM). Absorbance at 450 nm indicates a dose-dependent reduction in viability in parental cells, which was significantly attenuated in St-EPO cells. (**B**) Representative flow cytometry plots illustrating cell viability and death using PI staining. Cells were exposed to high-dose iron stress (0, 1000, and 2000 µM FAC) to evaluate the robust protective capacity of the EPO-R76E variant. Red indicates live cells, while blue indicates early and late apoptotic or dead cells. (**C**) Quantitative analysis of the percentage of PI-positive (dead) cells. Treatment with 1000 and 2000 µM FAC induced substantial cell death in parental ARPE-19, whereas St-EPO cells exhibited marked resistance to iron-induced lethality. Data are presented as mean ± SEM (*n* = 5 from 3 independent experiments). Statistical significance was determined using Student’s *t*-test ( * *p* < 0.05; ** *p* < 0.01, *** *p* < 0.001, **** *p* < 0.0001).

In parental ARPE-19 cells, FAC exposure resulted in a dose-dependent reduction in metabolic activity as measured by the WST-1 assay (Figure 1A). Conversely, St-EPO cells exhibited significantly higher viability across all concentrations, maintaining robust metabolic function even at 1000 µM FAC. To define the mode of cell death, we performed flow cytometry using PI staining. Parental cells subjected to iron stress showed a dramatic shift toward the PI-positive population, indicating the terminal membrane rupture associated with ferroptotic collapse [30]. St-EPO cells demonstrated a significant reduction in PI labeling (Figure 1B,C), confirming that EPO-R76E raises the threshold for iron-induced lethality. These results demonstrate that the cytoprotective efficacy of EPO-R76E, previously established in superoxide-driven models, extends to iron-dependent ferroptotic injury.

### 3.2. Restriction of the LIP and Modulation of Ferritin

**Figure 2:**
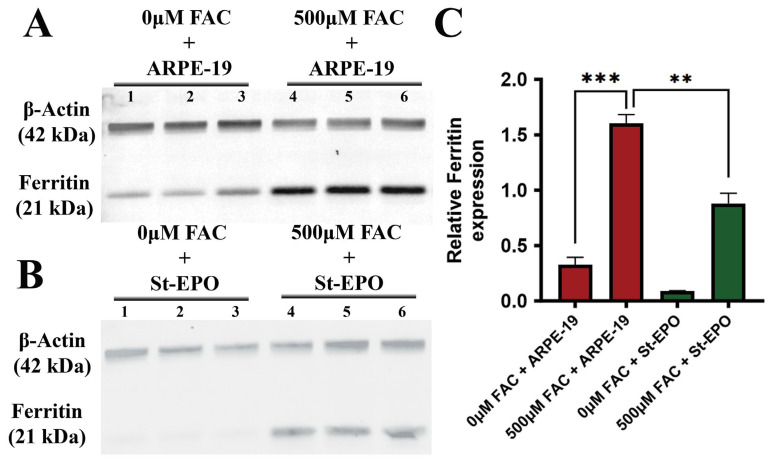
EPO-R76E reduces ferritin expression in ARPE-19 cells. (**A**) Representative immunoblots showing ferritin expression in parental ARPE-19 cells treated with or without 500 µM FAC. (**B**) Representative immunoblots showing ferritin expression in cells stably expressing the non-erythropoietic EPO-R76E variant (St-EPO), treated with or without 500 µM FAC. β-actin was used as the loading control. (**C**) Densitometric quantification of ferritin levels normalized to β-actin. Data are presented as mean ± SEM (*n* = 3). Statistical significance: ** *p* < 0.01; *** *p* < 0.001.

**Figure 3:**
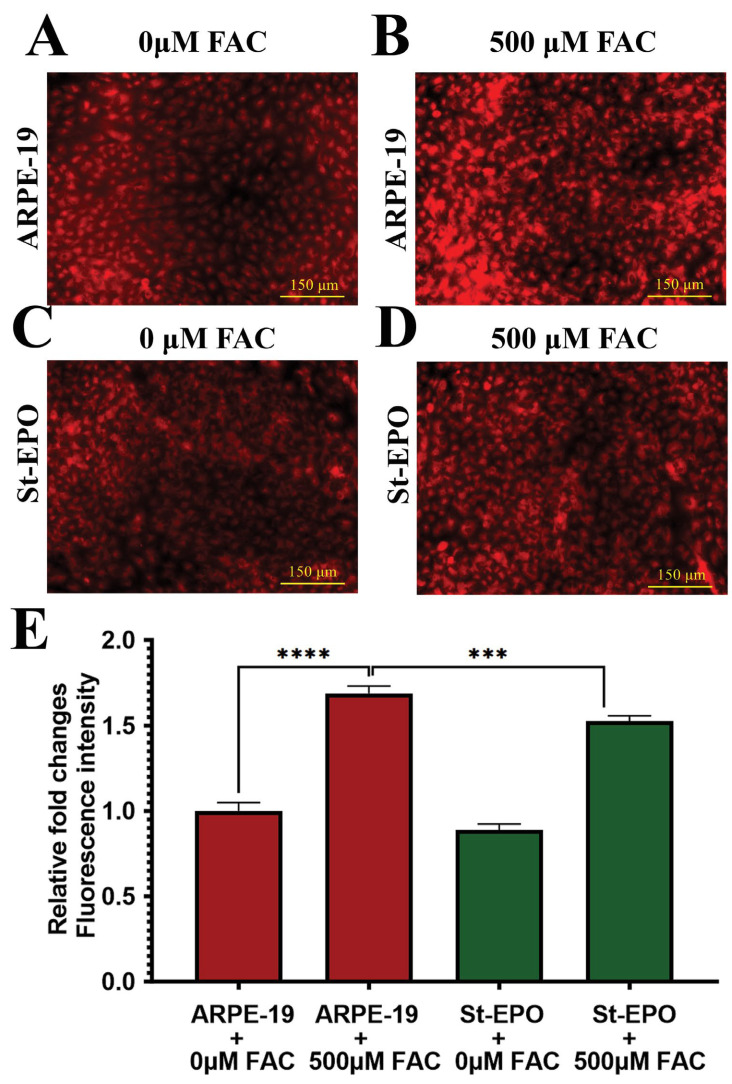
EPO-R76E decreases the LIP in ARPE-19 cells. Assessment of intracellular labile ferrous iron (Fe^2+^) using the Ferro-Orange fluorescent probe. Representative fluorescence images show: (**A**) untreated control ARPE-19 cells (0 µM FAC), (**B**) ARPE-19 cells treated with 500 µM FAC, (**C**) untreated control St-EPO cells (0 µM FAC), and (**D**) St-EPO cells treated with 500 µM FAC. FAC-treated parental ARPE-19 cells (**B**) exhibited intense orange fluorescence, indicating increased accumulation of labile iron, whereas this increase was markedly reduced in FAC-treated St-EPO cells (**D**). (**E**) Relative fluorescence intensity was quantified using ImageJ to assess intracellular Fe^2+^ levels. Data are presented as mean ± SEM (*n* = 3). Scale bar = 150 µm. Statistical significance: *** *p* < 0.001; **** *p* < 0.0001.

The initiation of ferroptosis is critically dependent on the expansion of the LIP, where redox-active ferrous iron (Fe^2+^) drives the production of hydroxyl radicals through Fenton chemistry [31]. Western blot analysis revealed a significant downregulation of Ferritin in St-EPO cells compared to wild-type controls under FAC challenge (Figure 2A–C), with a mean intensity level of 0.88 ± 0.16 AU. While Ferritin expression typically scales with iron load to minimize toxicity, the reduced levels in St-EPO cells, coupled with lower Ferro-Orange intensity, suggest that EPO-R76E promotes a more efficient state of intracellular iron homeostasis or reduced uptake. Utilizing the (Fe^2+^) sensitive fluorescent probe Ferro-Orange, we observed that FAC treatment triggered a massive intracellular accumulation of ferrous iron in parental cells (Figure 3A–E), with a mean intensity level of 1.52 ± 0.02 arbitrary units (AU). Notably, this accumulation was markedly attenuated in St-EPO cells. By limiting the availability of catalytic Fe^2+^, EPO-R76E effectively intercepts the ferroptotic cascade at the primary “trigger” stage.

### 3.3. Suppression of Lipid Peroxidation and Global ROS Burden

The definitive biochemical hallmark of ferroptosis is the catastrophic peroxidation of membrane polyunsaturated fatty acids (PUFAs) [32]. We employed the Click-iT™ Lipid Peroxidation imaging assay, which utilizes the incorporation of linoleamide alkyne (LAA) to visualize oxidized lipid species. FAC-treated parental cells displayed intense, widespread fluorescence, signaling severe membrane compromise (Figure 5A–E), with a mean signal intensity of 1.04 ± 0.12. In contrast, St-EPO cells showed significantly quenched signals, demonstrating that EPO-R76E expression prevents the propagation of lipid radical chain reactions. These findings were further supported by DCFDA analysis, which showed that the overall intracellular reactive oxygen species (ROS) burden was significantly lower in St-EPO cells (Figure 8A–E), with a mean level of 1.27 ± 0.15. This dual suppression of specific lipid peroxides and global ROS confirms that EPO-R76E functions as a multimodal antioxidant shield, protecting the RPE from the feed-forward oxidative loop that characterizes ferroptotic death.

### 3.4. Activation of the NRF2 Axis and Preservation of the GPX4 Shield

**Figure 4:**
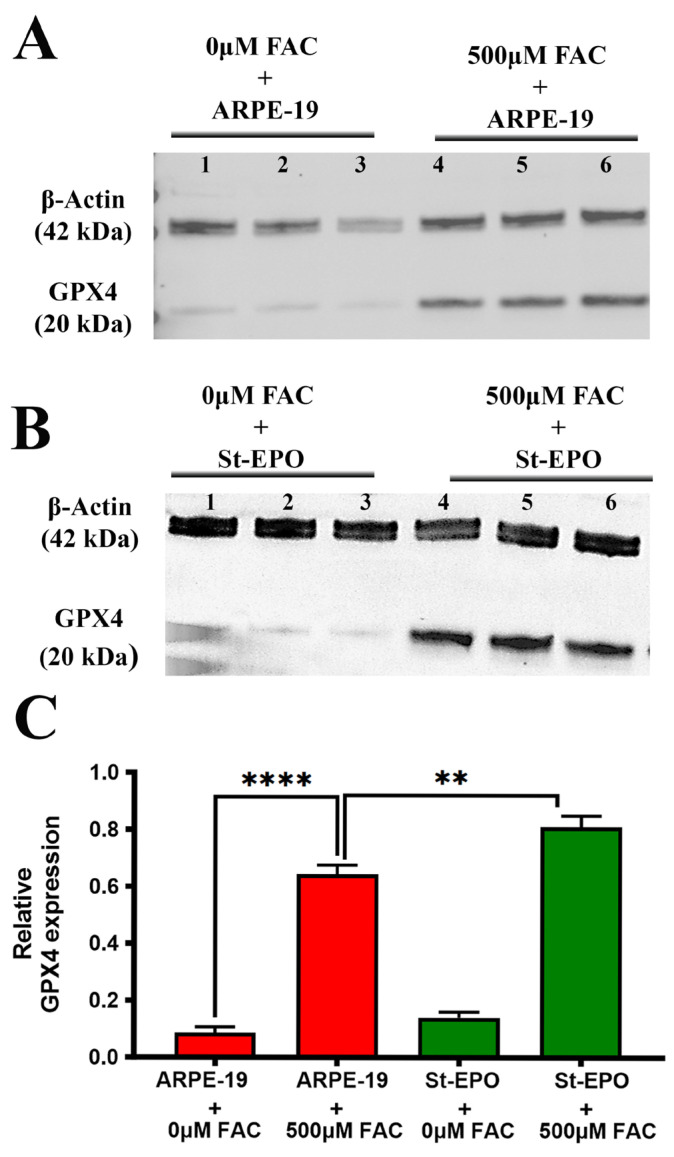
EPO-R76E upregulates GPX4 expression in ARPE-19 cells. EPO-R76E bolsters antioxidant defenses by upregulating GPX4 in ARPE-19 cells. (**A**) Representative immunoblots showing GPX4 expression in parental ARPE-19 cells treated with or without 500 µM FAC. (**B**) Representative immunoblots showing GPX4 expression in cells stably expressing the non-erythropoietic EPO-R76E variant (St-EPO), treated with or without 500 µM FAC. β-actin was used as the loading control. (**C**) Densitometric quantification of GPX4 protein levels normalized to β-actin. Data are presented as mean ± SEM (*n* = 3 independent experiments). Statistical significance was determined using Student’s *t*-test (** *p* < 0.01; **** *p* < 0.0001).

To identify the transcriptional drivers of this protection, we focused on the NRF2-antioxidant response element (ARE) pathway, which regulates the cellular defense against electrophilic stress [33]. RT-qPCR analysis revealed that EPO-R76E expression induced a coordinated upregulation of NRF2 and its key downstream targets, including CAT, NQO1, HO-1, and GSTM1 (Figure 7, Table 1). This transcriptional priming had direct consequences for the GPX4 enzyme, the primary orchestrator of ferroptosis resistance [34]. While iron overload typically depletes GPX4 protein through oxidative degradation, St-EPO cells significantly maintained GPX4 protein expression levels compared to parental cells (Figure 4A–C), with relative intensity levels of 0.86 ± 0.08 arbitrary units (AU). The preservation of the GPX4 shield, powered by an enhanced NRF2 antioxidant program, provides a mechanistic explanation for the observed resistance to lipid peroxidation and aligns with our established *in vivo* observations in Sod2-deficient mouse models [21].

**Figure 5:**
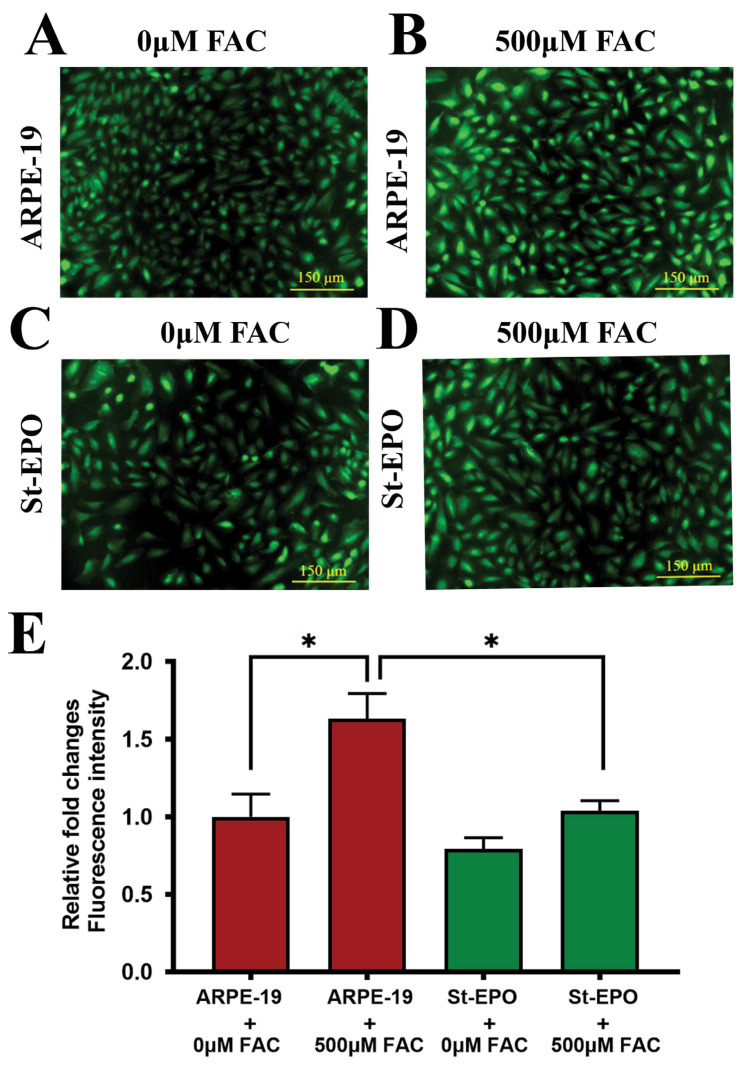
EPO-R76E suppresses lipid peroxidation in ARPE-19 cells. Assessment of lipid peroxidation using Click-iT™ fluorogenic imaging. Representative fluorescence images show: (**A**) untreated control ARPE-19 cells (0 µM FAC), (**B**) ARPE-19 cells treated with 500 µM FAC, (**C**) untreated control St-EPO cells (0 µM FAC), and (**D**) St-EPO cells treated with 500 µM FAC. Strong green fluorescence in FAC-treated parental cells (**B**) indicates high levels of lipid peroxidation, which was markedly attenuated in FAC-treated St-EPO cells (**D**). (**E**) Relative fluorescence intensity quantified via ImageJ. Data are expressed as mean ± SEM (*n* = 3). Scale bar: 100 µm. Statistical significance was determined by Student’s *t*-test (* *p* < 0.05).

### 3.5. Enhanced Autophagic Flux and Proteostatic Quality Control

**Figure 6:**
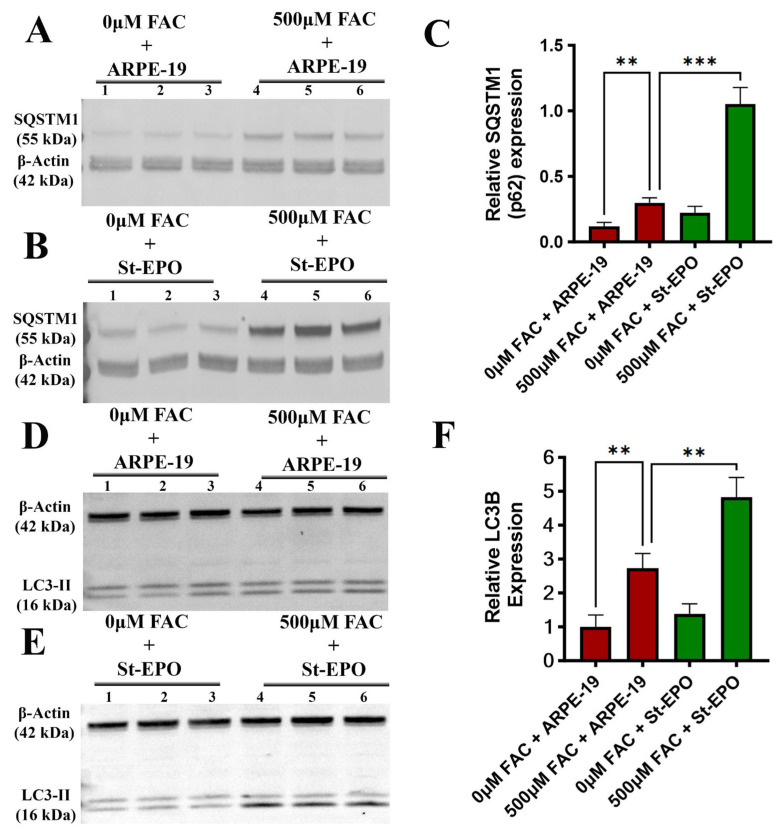
EPO-R76E modulates autophagic signaling by upregulating SQSTM1 and LC3B-II in ARPE-19 cells. Representative immunoblots of (**A**,**B**) SQSTM1 and (**D**,**E**) LC3B expression in parental ARPE-19 cells and those stably expressing the non-erythropoietic EPO-R76E variant (St-EPO), treated with or without 500 µM FAC. Β-ACTIN was used as an internal loading control. Densitometric quantification of (**C**) SQSTM1 and (**F**) LC3B-II protein levels were performed and normalized to β-actin. Data are presented as mean ± SEM (*n* = 3). Statistical significance was determined using Student’s *t*-test (** *p* < 0.01; *** *p* < 0.001).

**Figure 7:**
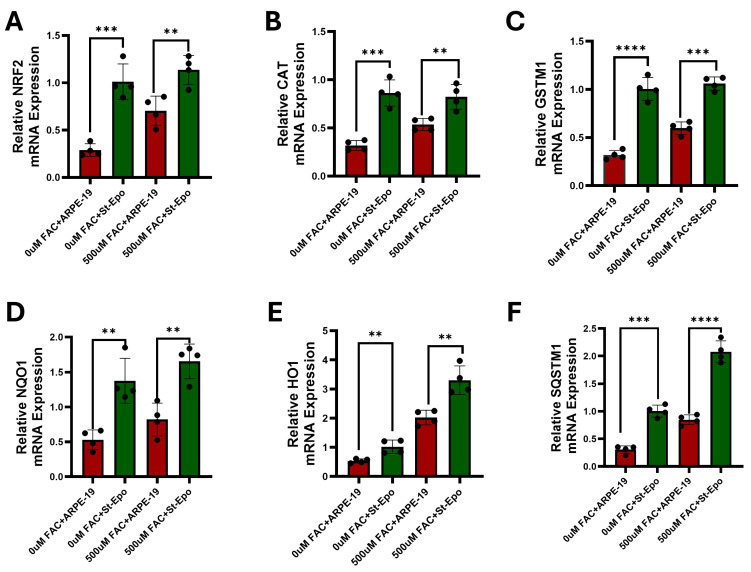
EPO-R76E induces a coordinated antioxidant transcriptional response in ARPE-19 cells. Quantitative real-time PCR (qRT-PCR) was employed to evaluate the mRNA expression of key antioxidant and phase II detoxification genes. Relative transcript levels are shown for (**A**) NRF2, (**B**) CAT, (**C**) GSTM1, (**D**) NQO1, (**E**) HO1, and (**F**) SQSTM1. Compared to parental ARPE-19 controls, stable expression of the non-erythropoietic EPO-R76E variant resulted in a robust and significant upregulation across the entire antioxidant gene panel. These data suggest that EPO-R76E primes the retinal pigment epithelium against oxidative stress via the NRF2 signaling axis. Data represents SEM (*n* = 3). Statistical significance was determined by Student’s *t*-test (** *p* < 0.01; *** *p* < 0.001; **** *p* < 0.0001).

Finally, we examined the impact of EPO-R76E on the autophagic machinery, which is essential for the clearance of damaged organelles and oxidized aggregates in the RPE [35,36,37,38]. We observed a robust induction of the autophagy adaptor SQSTM1/p62 at both the mRNA (2.081 ± 0.211) and protein intensity levels (1.052 ± 0.100 AU) in St-EPO cells (Figure 6A–C and Figure 7C). Furthermore, EPO-R76E facilitated the conversion of LC3B-I to LC3B-II, indicating active autophagosome formation and enhanced autophagic flux (Figure 6D–F). LC3B-II levels were significantly elevated (5.16 ± 0.91 AU), further supporting enhanced autophagic activity. The activation of autophagy by EPO-R76E suggests a multi-layered defense strategy: while the NRF2-GPX4 axis prevents the formation of damage, the autophagic machinery ensures the efficient clearance of pre-existing oxidative debris. This integrated approach, addressing iron homeostasis, antioxidant signaling, and proteostatic flux, provides a comprehensive framework for the cytoprotective efficacy of EPO-R76E in retinal degenerative environments.

**Figure 8:**
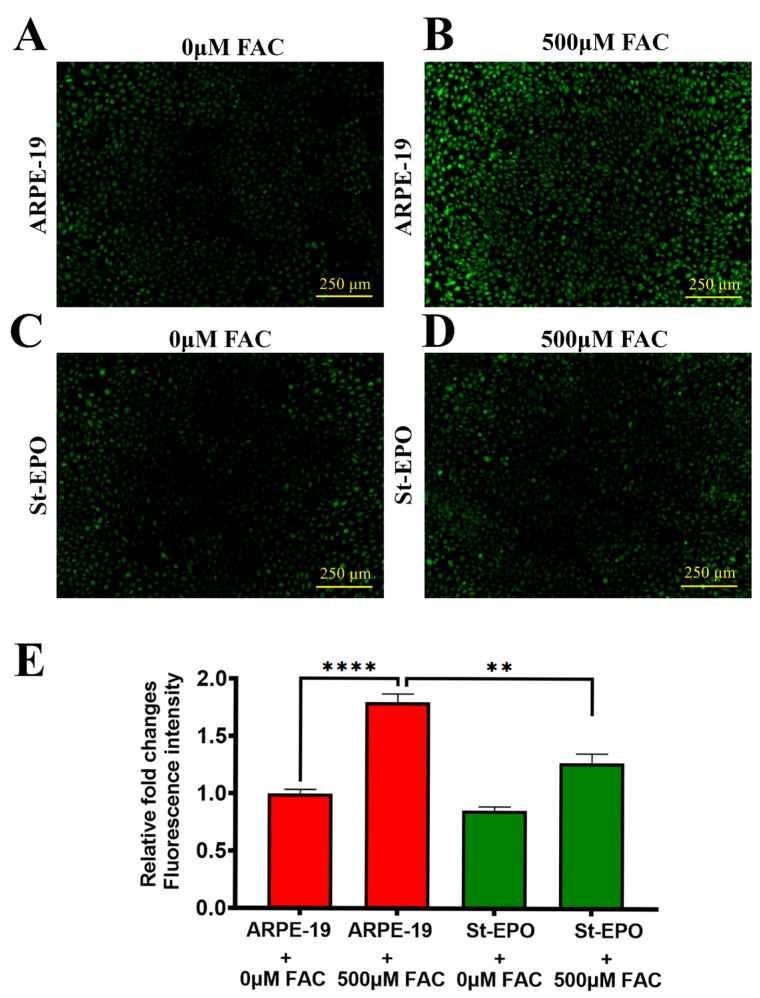
EPO-R76E attenuates iron-induced oxidative stress by reducing reactive oxygen species (ROS) accumulation in AR-PE-19 cells. Representative fluorescence images of intracellular ROS levels, visualized via DCFDA staining in parental ARPE-19 and EPO-R76E-stably expressing (St-EPO) cells following 24 h treatment with or without 500 µM FAC. (**A**) untreated control ARPE-19 cells (0 µM FAC), (**B**) ARPE-19 cells treated with 500 µM FAC, (**C**) untreated control St-EPO cells (0 µM FAC), and (**D**) St-EPO cells treated with 500 µM FAC. Parental cells exhibited a robust increase in DCFDA fluorescence following iron exposure, indicative of elevated oxidative burden, which was markedly suppressed in St-EPO cells. (**E**) Quantitative analysis of DCFDA fluorescence intensity performed using ImageJ. Data are expressed as mean ± SEM (*n* = 3). Scale bar: 100 µm. Statistical significance was determined using an unpaired two-tailed Student’s *t*-test (** *p* < 0.01; **** *p* < 0.0001).

## 4. Discussion

**Figure 9:**
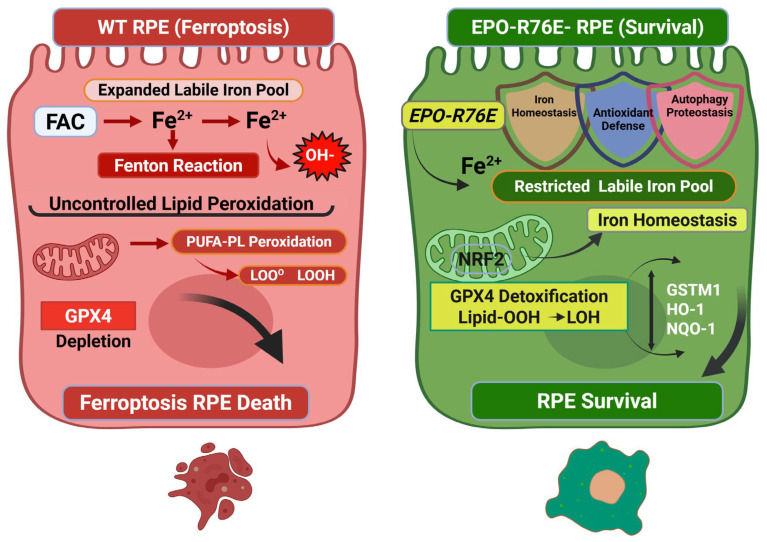
Schematic Model of EPO-R76E-Mediated Protection Against Ferroptosis in the RPE. Proposed mechanistic model illustrating the divergent cellular fates of parental and St-EPO ARPE-19 cells under iron-induced stress. (**Left**) (Ferroptosis Execution): In parental RPE cells, exposure to FAC triggers an expansion of the LIP. Excess catalytic ferrous iron (Fe^2+^) drives the production of hydroxyl radicals via Fenton chemistry, leading to the catastrophic peroxidation of membrane polyunsaturated fatty acids (PUFA-PL). The depletion of the GPX4 antioxidant shield allows for the unchecked propagation of lipid peroxyl radicals (LOO•), resulting in terminal membrane rupture and ferroptotic cell death. (**Right**) (EPO-R76E-Mediated Defense): In St-EPO cells, the stable expression of the non-erythropoietic EPO-R76E variant (delivered via an EF1-driven lentiviral vector) initiates a multimodal defense program. 1. Iron Sequestration: EPO-R76E restricts the LIP, effectively neutralizing the primary trigger of the Fenton reaction. 2. NRF2-GPX4 Axis: EPO-R76E promotes the nuclear translocation of NRF2, upregulating a coordinated antioxidant transcriptomic profile (including GSTM1, HO-1, and NQO1) and preserving GPX4 protein levels to quench lipid peroxidation. 3. Autophagic Flux: Enhanced p62/LC3B-mediated autophagy facilitates the clearance of oxidative debris and damaged organelles, maintaining RPE proteostasis and preventing cell death.

The preservation of the retinal pigment epithelium (RPE) is a primary therapeutic objective in the management of age-related macular degeneration (AMD). In the present study, we demonstrate that the non-erythropoietic erythropoietin variant, EPO-R76E, provides protective effects against iron-induced ferroptosis in ARPE-19 cells. Our results indicate that EPO-R76E acts through a multimodal mechanism by simultaneously restricting the labile iron pool, reinforcing the NRF2–antioxidant axis, and promoting autophagic flux, while avoiding the hematological side effects associated with wild-type EPO. A Schematic Model of EPO-R76E-Mediated Protection Against Ferroptosis in the RPE is shown in Figure 9.

The therapeutic potential of EPO-R76E in the retina is supported by a growing body of evidence. We previously demonstrated that EPO-R76E effectively rescues ARPE-19 cells from acute oxidative insults [20] induced by Paraquat, suggesting that its cytoprotective signaling is broadly applicable across different oxidative triggers. Furthermore, the translational relevance of this variant was validated in a complex animal model of RPE-specific Sod2 deletion, where EPO-R76E gene therapy significantly preserved RPE integrity and visual function [21]. The current study expands upon these findings by specifically identifying ferroptosis as a key pathological target, providing a missing mechanistic link between iron dyshomeostasis and the RPE degeneration observed in our previous Sod2 knockout models.

A hallmark of ferroptosis is the iron-catalyzed accumulation of lipid hydroperoxides. In the aging RPE, the accumulation of iron often termed “retinal siderosis” fuels the Fenton reaction, generating hydroxyl radicals that initiate the catastrophic peroxidation of polyunsaturated fatty acids [39]. Our study utilized the Ferro-Orange probe to show that EPO-R76E expression significantly attenuates the LIP following FAC challenge (Figure 2). This is a critical observation: while many studies on EPO focus on anti-apoptotic signaling, our data suggests a more direct role in iron homeostasis. By reducing the LIP, EPO-R76E “quenches” the Fenton chemistry at its source. This reduction in labile iron directly correlates with the suppression of lipid radical propagation, as evidenced by the Click-iT™ and DCFDA assays (Figure 3 and Figure 6). This supports the notion that modulating the LIP is a viable strategy for controlling oxidative damage and cell survival in retinal tissues [40].

One of the most significant mechanistic insights from our research is the robust activation of the NRF2-antioxidant axis. NRF2 is the master regulator of the antioxidant response element (ARE), and its activity is known to decline in the aging RPE. We found that EPO-R76E induced a coordinated transcriptional program, upregulating NRF2, CAT, NQO1, and GSTM1 (Figure 5). This transcriptional reinforcement is functionally linked to the preservation of GPX4 protein levels (Figure 3). GPX4 is the primary enzyme responsible for neutralizing lipid hydroperoxides, and its depletion is a defining step in the ferroptotic cascade [41]. While iron overload typically depletes GPX4, EPO-R76E expression partially restored it. This suggests that EPO-R76E does not just block damage; it “primes” the cell’s endogenous repair capacity, a strategy that has shown success in other models of RPE oxidative stress [42].

A novel dimension of our study is the link between EPO-R76E and autophagic flux, evidenced by the induction of SQSTM1/p62 and LC3B-II conversion (Figure 4). In the high-metabolic environment of the RPE, autophagy is vital for clearing damaged organelles and oxidized protein aggregates that would otherwise contribute to drusen formation [43]. While some studies suggest that excessive “ferritinophagy” can promote ferroptosis by degrading ferritin [44], our data suggests that EPO-R76E promotes a balanced, protective autophagic response. By enhancing the clearance of “oxidative debris,” EPO-R76E ensures that the intracellular environment remains optimized for survival. This multimodal approach represents a more comprehensive therapeutic strategy than single-target antioxidants, which have often failed to show efficacy in clinical trials for AMD.

We chose the ARPE-19 cell line as our experimental foundation, as it provides a consistent and well-characterized platform for untangling the complex molecular signaling that governs RPE health [25,45,46,47,48,49,50]. ARPE-19 cells are particularly valued for their ability to model RPE-specific responses to oxidative stress and iron-mediated injury, as established in numerous mechanistic studies investigating AMD pathogenesis [42,51]. While ARPE-19 cells may not perfectly mirror the post-mitotic, polarized state of the *in vivo* RPE-often exhibiting more mesenchymal-like characteristics when sub-confluent-they remain the gold standard for high-throughput molecular dissection and gene therapy validation [52,53].

We acknowledge that our FAC-induced iron overload model represents an acute challenge. However, this model accurately reflects the biochemical stressors and increased LIP present in the subretinal space of AMD patients [54,55]. Furthermore, recent comparative studies have confirmed that FAC treatment in ARPE-19 cells effectively mimics the ferroptotic and senescent phenotypes observed in aging RPE [9,45]. While we are encouraged by our established success in the *Sod2* mouse model [21], we recognize that the next vital step involves moving into more complex human models. Future work using primary human RPE, induced pluripotent stem cell-derived retinal pigment epithelium (iPSC-RPE) or 3D retinal organoids [56,57,58] will be essential to truly bridge the gap between our current findings and clinical application, ensuring that the efficacy and safety of EPO-R76E translate effectively to the human eye.

While our data highlights a clear intersection between EPO-R76E signaling and the GPX4-mediated antioxidant shield, we recognize that GPX4 is part of a much larger, integrated defense network. We did not directly quantify other canonical ferroptotic regulators, such as ACSL4, the primary driver of PUFA-phospholipid synthesis [59], or FSP1, the CoQ10-dependent parallel defense pathway [60]. Recent evidence specifically in RPE models suggests that both the FSP1 and GPX4 pathways act as essential, independent checkpoints for inhibiting retinal ferroptosis [12]. Given that EPO-R76E so robustly attenuated lipid peroxidation and preserved GPX4 levels in our study, it is highly probable that these parallel systems are also being recruited or bolstered. Moving forward, it will be vital to define the precise influence of EPO-R76E on the ACSL4-dependent lipid remodeling environment [61], as this likely dictates how sensitive the RPE remains to ferroptotic stimuli in a chronic disease state.

It is important to note that, in the present study, ferroptosis was inferred based on established biochemical and molecular hallmarks, including lipid peroxidation, reactive oxygen species generation, iron accumulation, and GPX4 modulation. However, definitive confirmation using gold-standard approaches-such as ferroptosis-specific inhibitors (e.g., ferrostatin-1 or liproxstatin-1), rescue experiments, or ultrastructural validation by transmission electron microscopy-was not performed. Therefore, while our data strongly supports ferroptosis-associated processes, future studies incorporating these complementary approaches will be essential to conclusively validate ferroptosis and further strengthen the mechanistic insights.

Our findings indicate that EPO-R76E is associated with modulation of the NRF2–GPX4 axis, preservation of GPX4, and activation of autophagic flux; however, these observations primarily represent correlative relationships rather than established mechanistic dependencies. In the present study, pathway-specific inhibition or genetic validation approaches were not performed due to scope limitations. Therefore, the proposed mechanistic interpretations should be considered in this context, and further functional studies are warranted to define the causal contribution of these pathways to the protective effects of EPO-R76E against ferroptosis.

A limitation of this study is the use of ARPE-19 cells as an in vitro model of retinal pigment epithelium (RPE). Although ARPE-19 cells are widely used due to their practicality and ease of culture, they do not fully replicate the phenotype, polarity, and gene expression profile of primary RPE cells. Notably, differences in differentiation status and certain functional properties may influence experimental outcomes. Therefore, validation in primary RPE cells and/or *in vivo* models would further strengthen the physiological relevance of the findings.

## 5. Conclusions

In conclusion, our study identifies EPO-R76E as a potent therapeutic candidate for the treatment of RPE degeneration. By simultaneously modulating the labile iron pool, reinforcing the NRF2-antioxidant network, and activating autophagic quality control, EPO-R76E provides a “multimodal” defense against ferroptosis. Given its favorable safety profile and demonstrated efficacy across both in vitro and *in vivo* models of oxidative injury, EPO-R76E-based gene therapy offers a promising approach to preserve vision in patients with iron-associated retinal diseases.

## Figures and Tables

**Table 1:** Primer Sequences for RT-qPCR Analysis.

Gene	Forward Primer (5′ → 3′)	Reverse Primer (5′ → 3′)	Size (bp)
*GSTM1*	TTCAAGCTGGGCCTGGACTT	TCTGGATTGTAGCAGATCATGCCC	148
*HO-1*	TGCACACCCAGGCAGAGAAT	GTGTGTAGGGGATGACCTCCTG	172
*NRF2*	CATGCCCTCACCTGCTACTT	TGTTCTGGTGATGCCACACT	162
*CAT*	TGGAGCTGGTAACCCAGTAGG	CCTTTGCCTTGGAGTATTTGGTA	138
*NQO1*	GAAGAGCACTGATCGTACTGGC	GGATACTGAAAGTTCGCAGGG	156
*SQSTM1*	CAGCTGTTTCGTCCGTACCT	CCATCCTCATCGCGGTAGTG	135
*β-ACTIN*	GCTATCCCTGTACGCCTCTG	CCATCTCTTGCTCGAAGTCC	194

## Data Availability

The original contributions presented in this study are included in the article/Supplementary Material. Further inquiries can be directed to the corresponding author.

## Supplementary Materials

The following supporting information can be downloaded at: https://www.mdpi.com/article/10.3390/antiox15050647/s1, Figure S1: Re-validation of Stable EPO-R76E Expression and Plasmid Design. (A). Schematic diagram of the EPO R76E expression plasmid used for lentiviral transduction. The construct encodes human EPO R76E followed by a self-cleaving T2A peptide and α-tubulin reporter gene under the control of a EF1 promoter. (B). Confirmation of T2A (~26 kDa)-mediated co-expression via ~51 kDa α-tubulin immunoblotting. (C). Western blot showing EPO-R76E expression in lysates from ARPE-19 and transduced (ARPE-19-EPO-R76E) cells. EPO (~26 kDa) is detected only in transduced cells. β-actin (~42 kDa) serves as a loading control. [M denotes the molecular weight standards, C represents ARPE-19 control cells, and EPO indicates ARPE-19 cells stably expressing EPO-R76E]. (D) RT-qPCR verification of significantly elevated EPO mRNA transcripts in St-EPO cells (*n* = 3) (**** *p* < 0.0001).

## Abbreviations

The following abbreviations are used in this manuscript:

RPE	Retinal Pigment Epithelium
AMD	Age-Related Macular Degeneration
ACSL4	Acyl-CoA Synthetase Long-Chain Family Member 4
EPO-R76E	Modified variant of Erythropoietin
GPX-4	Glutathione peroxidase 4
ROS	Reactive Oxygen Species
LIP	Labile Iron Pool
NRF2	Nuclear factor erythroid 2-related factor 2
DCFDA	2′,7′-dichlorofluorescin diacetate
St-EPO	ARPE-19 cells expressing EPO-R76E
HO-1	Heme oxygenase -1
GSTM1	Glutathione S-Transferase Mu 1
NQO-1	NAD(P)H quinone dehydrogenase 1
CAT	Catalase
FAC	Ferric Ammonium Citrate
A. U.	Arbitrary Unit
PUFAs	Polyunsaturated fatty acids
4-HNE	4-hydroxynonenal
FSP-1	Ferroptosis Suppressor Protein 1
MDA	Malondialdehyde
SQSTM1	Sequestosome-1
LC3B	Microtubule-associated protein 1 light chain 3B
ARE	Antioxidant Response Element
RT-qPCR	Reverse Transcription–quantitative Polymerase Chain Reaction
iPSC-RPE	Induced Pluripotent stem cell-derived Retinal Pigment Epithelium
LAA	Linoleamide Alkyne
VEGF	Vascular Endothelial Growth Factor
