# Supplementary material for: Non-Erythropoietic EPO (EPO-R76E) Protects RPE Cells from Ferroptosis by Modulating the Labile Iron Pool and NRF2-GPX4 Axis
Source: Antioxidants (Basel). 2026 May 20;15(5):647. doi: 10.3390/antiox15050647 (PMC13203161; doi:10.3390/antiox15050647)
Supplement: Supplementary file 1 [file antioxidants-15-00647-s001.zip › Supplementary Figure S1.pdf]

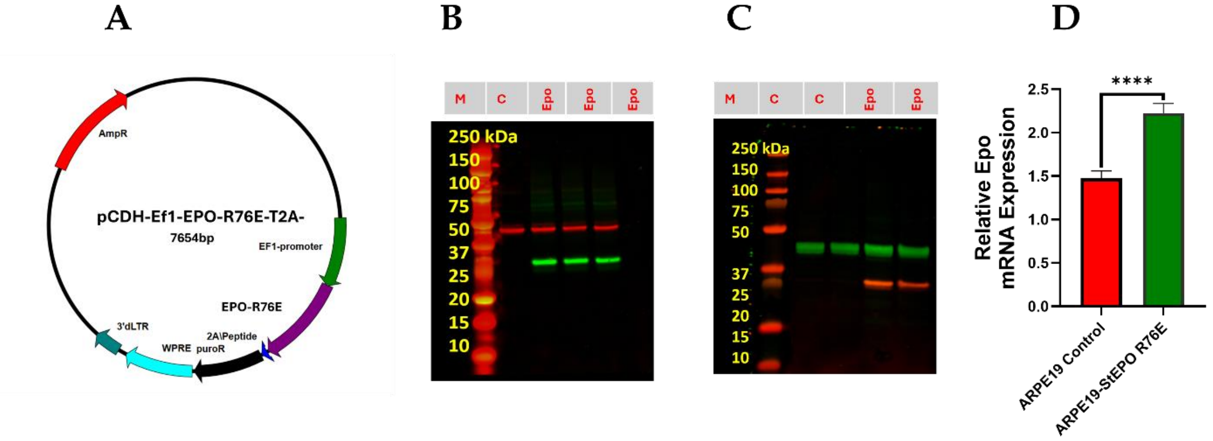

Supplementary Figure S1. Re-validation of Stable EPO-R76E Expression and Plasmid Design. (A). Schematic diagram of the EPO R76E expression plasmid used for lentiviral transduction. The construct encodes human EPO R76E followed by a self-cleaving T2A peptide and α-tubulin reporter gene under the control of a EF1 promoter. (B). Confirmation of T2A (~26 kDa)-mediated co-expression via ~51 kDa α-tubulin immunoblotting. (C). Western blot showing EPO-R76E expression in lysates from ARPE-19 and transduced (ARPE-19-EPO-R76E) cells. EPO (~26 kDa) is detected only in transduced cells. β-actin (~42 kDa) serves as a loading control. [M denotes the molecular weight standards, C represents ARPE-19 control cells, and EPO indicates ARPE-19 cells stably expressing EPO-R76E]. (D) RT-qPCR verification of significantly elevated EPO mRNA transcripts in St-EPO cells (n=3) (****$p < 0.0001$).
